# Corrigendum to “Valorization of Oued Sebou Natural Sediments (Fez-Morocco Area) as Adsorbent of Methylene Blue Dye: Kinetic and Thermodynamic Study”

**DOI:** 10.1155/2020/4815767

**Published:** 2020-09-30

**Authors:** Abdelaziz Dra, Karim Tanji, Abdellah Arrahli, El Mustafa Iboustaten, Abdelali El Gaidoumi, Achaimae Kherchafi, Aziz Chaouni Benabdallah, Abdelhak Kherbeche

**Affiliations:** ^1^Laboratory of Catalysis, Materials and Environment, Higher School of Technology, Sidi Mohamed Ben Abdellah University of Fez, Fez 30000, Morocco; ^2^Euromed Research Center, National Institute of Applied Sciences, Euro-Mediterranean University of Fez, Fez, Morocco; ^3^Higher School of Technology of Khenifra, Sultan Moulay Slimane University, Beni Mellal, Morocco

In the article titled “Valorization of Oued Sebou Natural Sediments (Fez-Morocco Area) as Adsorbent of Methylene Blue Dye: Kinetic and Thermodynamic Study” [1], the author Abdellah Arrahli was affiliated to “Laboratory of Catalysis, Materials and Environment, Higher School of Technology, Sidi Mohamed Ben Abdellah University of Fez, 30000 Fez, Morocco,” which is incorrect. The correct affiliation for this author is as follows:

  Euromed Research Center, National Institute of Applied Sciences, Euro-Mediterranean University of Fez, Morocco.
  E-mail address: a.arrahli@insa.ueuromed.org.

The corrected list of affiliations is shown in the author information above.
